# Mobility in informal settlements during a public lockdown: A case study in South Africa

**DOI:** 10.1371/journal.pone.0277465

**Published:** 2022-12-22

**Authors:** Yael Borofsky, Isabel Günther

**Affiliations:** Department of Humanities, Social and Political Sciences, Development Economics Group, ETH Zurich, Zurich, Switzerland; Columbia University, UNITED STATES

## Abstract

Many African countries quickly responded to the COVID-19 pandemic in 2020 with lockdowns of public life. Yet, many have large numbers of dense informal settlements where infrastructure is shared, houses are small, and residents live on low incomes. These conditions make complying with curfews extraordinarily difficult. Using pedestrian motion sensors installed throughout an informal settlement in Cape Town, South Africa, we study how the lockdown affected mobility in the evenings, early mornings, and during the nights between February 14 and June 18, 2020. We find that mobility was already decreasing in March prior to the start of lockdown by 23% in paths—about half of the overall decline—and by 19% in shared courtyards. Starting with the lockdown on March 27, pedestrian activity decreased by 48% in comparison to February 2020 across paths and by 61% in shared courtyards. We notice the biggest changes on weekends, normally key leisure times, and between 6:00 pm and 9:00 pm and between 6:00 am and 8:00 am, spanning typical commute hours, though these hours continue to have the most activity indicating some people continue to commute. The results show that mobility reduction is large, though generally smaller than reductions observed in high-income countries. We find that residents of informal settlements comply with state-mandated lockdowns to the best of their ability given the circumstances, but that awareness of COVID-19 with less strict regulations prior to lockdown also led to mobility declines.

## Introduction and background

As COVID-19 spread early in 2020, African countries were among the quickest to follow World Health Organization (WHO) guidelines and imposed strict lockdowns to limit mobility as well as most social interactions, even though many countries still had fewer than 100 cases at the time. South Africa, in particular, instated one of the strictest lockdowns in the world [1]. Despite the rapid response, many academics and thought leaders of civil society were almost as quick to point out that with so many low-income, dense informal settlements in cities throughout Africa, lockdowns may be, at best, impractical and infeasible and, at worst, more devastating than COVID-19, itself [2–11].

In the two years since the start of the COVID-19 pandemic, Gil et al. [12] finds that the economic impact of lockdowns on residents in informal settlements has indeed been substantial, particularly with regard to employment losses. In this study, we aim to better understand the concern about the feasibility of lockdowns in informal settlements by analyzing data from novel, nighttime pedestrian motion sensors (S1 Fig), which were installed in an informal settlement as part of a pre-existing study in Cape Town, South Africa. We analyze whether widespread concerns about non-compliance with curfew regulations in informal settlements are reflected in motion at night in this informal settlement.

Since roughly one in eight people worldwide live in informal settlements, knowing more about the feasibility of lockdowns and compliance with mobility restrictions in these neighborhoods is critical in a pandemic [13]. We use the term “informal settlement,” rather than “slum,” since “slum” can have a derogatory connotation [14]. There are several elements of life in these neighborhoods that make social distancing, let alone lockdowns of social and economic life, problematic [14, 15]. While informal settlements vary in terms of physical form, size, and infrastructure access, one major characteristic is that water and sanitation infrastructure are typically shared. Without access to private water and sanitation, people have to go out multiple times per day, making it impossible to follow strict curfews and avoid contact with non-household members.

Density is another major concern. While the number of household members sharing a living space varies, most informal homes are small. In the informal settlement we study, rooms are typically shared. With little indoor or private outdoor space, households do many daily activities, such as laundry, in public or semi-public spaces, such as pathways or shared space between homes.

The third major concern is economic. Many people living in informal settlements are low-wage earners in the informal sector, such as day workers or domestic workers, with little job security or social protection and small savings. Asking these people not to work often means asking them to forego income they need to purchase even basic necessities. For example, one survey of 19,000 South Africans finds that within two weeks of lockdown, two thirds of respondents living in poor, urban areas did not have enough money for food [16]. It is unrealistic to expect that people living in this precarious situation will not continue to search for and travel to available work.

As a result, many researchers, NGOs, and health care providers worried that residents in informal settlements were not reducing mobility and social contact in response to restrictions, which are essential to limit the spread of COVID-19 (in the absence of a vaccine) and other future highly infectious diseases. Several media reports from South Africa early in the pandemic depicted residents of informal settlements outdoors, sometimes without masks or in close proximity to others, when the law stipulated that everyone must be inside [17, 18]. Two hypotheses have emerged from this discourse to explain how curfews might affect the behavior of residents and the spread of COVID-19 in low-income neighborhoods. First, the “higher mobility” hypothesis is that lockdowns have less of an impact on mobility in lower-income communities either because low-income people are more likely be essential workers, less likely to have the option of working remotely, less likely to have the financial capacity to stay home without working, or less likely to comply with lockdowns for other reasons. Second, the “crowded housing” hypothesis argues that both small and dense housing and shared essential service infrastructure create an environment conducive to the spread of COVID-19, since limited space makes it difficult to stay indoors, while density and shared sanitation infrastructure make it difficult to maintain social distance from others [19]. While in 2022 it is now known that COVID-19 infection is much less likely outdoors, during the study period it was still recommended to maintain social distance outside, hence why we mention density as a contributor to the spread of COVID-19.

In response to these explanations, a rapidly growing body of literature seeks to better understand behavioral responses to the state-mandated lockdowns in lower-income countries, especially in urban areas, with mixed results. For example, phone survey data has provided some insight into self-reported activity patterns [6, 8, 10, 16, 20–22]. In a survey of more than 1,400 low-income, urban residents in Johannesburg and Accra conducted in April 2020, Durizzo et al. [8] find that 25–40% of people still report attending large gatherings and 10–20% report receiving guests at home. In contrast, a survey (N = 19,000) in South Africa run by the Human Sciences Research Council [16] finds that almost everyone reports complying with lockdown restrictions (staying home or only leaving for essentials). One study in Nairobi, Kenya targeting residents of informal settlements (N = 2,009) conducted early in the pandemic, finds that while almost all participants report staying home more, fears about income loss and food shortages are the main reason why respondents report that measures like quarantines or self-isolation would not be feasible [10].

In addition to self-report data for lower-income countries, for high- and middle-income countries many studies draw on large-scale data from Google, Apple, or nationally available mobile phone data to study mobility changes in response to lockdowns in higher-income countries. See, e.g., for the US: Chang et al. [23], Warren & Skillman [24], Klein et al. [25], Cronin & Evans [26], Coven & Gupta [27], Lee et al [28]; for Italy: Pepe et al. [29]; for France: Pullano [30]; for the UK: Jeffrey et al. [31]; for Spain: Aloi et al. [32]; for Brazil: Queiroz et al [33]; These studies overwhelmingly document mobility declines in response to both the pandemic, in general, and lockdowns specifically, with declines as high as 60–80% [24–26, 30, 32], but as low as 40–50% [28, 29]. A few studies use Google Mobility data to analyze lower-income countries [9, 34, 35]. Bharati & Fakir [34] find in a study of 117 countries (including both high-, middle-, and low-income countries) that stricter lockdowns in poorer countries reduce mobility more than in richer ones. In contrast, Bargain & Aminjonov [9] find for Egypt, Kenya, Nigeria, South Africa, Peru, Brazil, Argentina, Mexico, and Colombia that mobility for work decreases less in lower-income areas than in higher-income ones, while other activities show less of a discrepancy between richer and poorer areas. Last, three studies use mobile phone tracking apps to measure mobility in response to the lockdown. Two research groups in Switzerland [36, 37] each find a roughly 70% decrease in average daily distance (km) after lockdown began on March 16. For rural Thailand, Haddawy et al. [38] find mobility decreased by 90%.

Yet, to our knowledge, only one other study tries to measure mobility among residents from informal settlements in response to the global lockdown [19]. Using phone location data in Mumbai India, Sheng et al. [19] find little difference in the level of mobility between residents of informal settlements and those living in formal areas. Yet, they do not study mobility patterns within informal settlements, but rather the general mobility of residents from informal compared to formal neighborhoods out into the broader area. This insight is important, but it is only one piece of the complete picture describing how residents of informal areas responded to lockdown restrictions.

Importantly, individuals living in informal settlements may not necessarily be well represented in Google Mobility (or other types of phone) data [9, 19, 34]. First, as Sheng et al. [19] observe in Mumbai informal settlements, mobile phones are often shared. Second, in our setting, pre-paid cellular data is expensive and residents often switch off cellular data to control usage, only purchase WhatsApp data, or go for stretches with none at all. Third, mobility tracking apps are limited by GPS accuracy, making it hard to identify tracks within dense, informal neighborhoods.

We contribute a unique, hyper-local perspective to the growing number of studies on the impact of government lockdowns on mobility in a particularly difficult-to-study context—informal settlements. We use data from previously-installed nighttime pedestrian motion sensors in an informal settlement in Cape Town with about 2,300 residents (as of 2019) to analyze how nighttime activity patterns changed in response to South Africa’s lockdown, which began on March 27, 2020. Rather than tracking individuals, the sensors measure activity frequency (pedestrian count) in the areas where they are installed. Our data suggest that residents seem to comply with lockdown restrictions as much as possible, though not perfectly, and that many seem to have already reduced mobility in the weeks prior to the official lockdown, around when the first COVID-19 cases were reported in South Africa and a state of disaster was announced.

## Context of study site

The informal settlement we study is one of approximately 450 in the City of Cape Town, the second largest city in South Africa (we do not disclose the name due to ethical concerns) [39]. This thirty-year old informal settlement is home to approximately 2,300 residents and is located in a township called Khayelitsha that was zoned as Black African under apartheid. The path network inside the informal settlement is made up of paths, or through-routes, and private or semi-private shared compounds, like courtyards or cul-de-sacs. Compounds vary in terms of the number of households living inside, but they are similar in that the households have blocked all but one access path to their front doors and that access path is typically secured with a gate (which is sometimes locked at night). Therefore, the activity measured in compounds is more likely to be either the result of residents of the houses in that compound doing chores or socializing in the shared space, whereas activity measured in paths is more likely to be through-traffic from residents walking from one place to another. Residents living in paths and compounds all have similar levels of access to shared sanitation and communal water taps.

South Africa has had one of the most severe COVID-19 outbreaks in Africa [40, 41] and also instated one of the strictest lockdowns in the world in response to the first wave of the virus [1]. The first known case of COVID-19 was confirmed on March 3, 2020 in Kwazulu Natal and announced on March 5, 2020 [42]. On March 11^th^, the WHO formally announced that the COVID-19 outbreak constituted a pandemic [43]. On the same day, authorities confirmed the first case in the City of Cape Town, a city of approximately four million people, bringing the total number of cases in the country to 13. No mobility restrictions were yet recommended in Cape Town [44, 45].

On March 15, 2020, President Cyril Ramaphosa announced a national state of disaster. The country had 61 confirmed COVID-19 cases, some due to community transmission. President Ramaphosa implemented a travel ban on foreign nationals from high-risk countries, shut down 35 of 53 land ports and two sea ports, prohibited gatherings of 100 or more people, cancelled celebrations of upcoming national holidays, ordered schools to close on March 18, 2020, suspended visits to correctional facilities, called on businesses to put hygiene control measures in place, prohibited liquor sales after 6:00 pm, and limited the capacity of alcohol establishments [46, 47]. This announcement represented the first substantive call from the South African government for citizens to practice social distancing.

By March 21^st^, the Western Cape Premier Alan Winde announced 74 confirmed COVID-19 cases in the Western Cape, the province that includes Cape Town, and began directly encouraging people to stay home if possible and maintain a 1.5 meter distance from others in public [48]. By March 23,^,^ 2020, when President Ramaphosa announced a nationwide lockdown to start on March 27, the country had 402 confirmed cases [46]. Between March 27, 2020 and April 30, 2020, South Africa implemented what became known as a Level 5 lockdown–one of the strictest in the world (see S2 Fig). South Africans were not allowed to leave home unless they were essential workers, were going out to purchase essentials, like food and medicine, or were seeking healthcare, banking services, or government aid. On May 1^st^, South Africa moved from Level 5 to Level 4 lockdown, in which residents were allowed to go out from 6:00 am—9:00 am for recreation and an 8:00 pm to 5:00 am curfew was in effect. Level 3 began on June 1, 2020 and involved re-opening South Africa’s economy along with a relaxation of the limits on non-essential outdoor activity. Alcohol purchased for home consumption was allowed again. Gatherings (and funerals of more than 50 people) as well as all activities that involved large gatherings of people remained prohibited.

All Level 5 and 4 (March 27 until May 31, 2020) lockdown regulations could affect movement patterns, however, certain patterns were unlikely to change. Residents of informal settlements in South Africa, including the settlement we study, often share water and sanitation facilities, meaning that it would be nearly impossible to have perfect compliance with any curfew. People who became unemployed were no longer commuting. Thus, expected activity peaks during the morning and evening commuting period should be drastically lower. On the other hand, more unemployed people could also mean crowded households and thus, people may step out more at any hour to get fresh air or take a break from other household members. Durizzo et al. [8], for example, find that 17% of South Africans in their sample report that living in a crowded or single-room home is an obstacle to following lockdown regulations.

## Data and method

As part of a pre-existing community-based study focused on public lighting, we installed 171 proximity infrared (PIR) sensors on 121 paths and 50 private or semi-private shared compounds (like courtyards or cul-de-sacs) throughout an informal settlement in February 2020. The larger study was conducted in close collaboration with a Khayelitsha-based NGO and the leadership of the informal settlement. When fieldwork for the original project was interrupted in March 2020 by the COVID-19 pandemic, these sensors remained in place, passively gathering motion data, which presented an opportunity to examine how pedestrian activity in this community changed in response to the lockdown. Working individually, our field team collected these data using Bluetooth-enabled mobile phones during the three-hour window in which South Africans were permitted to be outdoors for non-essential purposes (Level 4).

The sensors were developed in collaboration with Sensen (http://www.sensen.co/), a company that builds dataloggers for international development projects. The PIR sensor detects differentials in thermal radiation, which trigger the device to record a count (S1 Fig). Every five minutes the sensor saves the trigger count in that five-minute period, recording no other information about passersby. Thus, our dataset includes a count for every five-minute period of each day that the sensors are installed, activated, and functional. The sensitivity of the PIR sensor prevents it from accurately measuring motion during the day, when heat created by the building materials common in Cape Town’s informal settlements (e.g., zinc or corrugated iron) causes false triggers. Therefore, we only study activity between 6:00 pm—8:00 am.

Unfortunately, sensor attrition was a problem, since dysfunctional sensors could not be repaired or replaced during the lockdown. The most common reasons for sensor attrition were battery discharge and vandalism/theft. Under normal circumstances our team can recharge batteries, however, under lockdown the restrictions on outdoor activities as well as health concerns for the field staff prevented maintenance. In addition, several sensors were stolen or damaged at the end of May. We removed all remaining sensors between June 19–20 in order to save them for the original study they were intended for (on hold due to the pandemic).

Using data from sensors that were active throughout the study period, we have 60 sensors in paths (50.4% attrition) and 26 sensors in compounds (52% attrition). We study changes in activity in response to the evolving COVID-19 pandemic in South Africa, and in response to both the Level 5 and Level 4 lockdowns between 6:00 pm and 8:00 am for the three-month period from February 14 –May 14, 2020. If we include all data until June 18, 2020 (the day before we began removing sensors), we have data from 21 path sensors and 18 compound sensors.

Since data is transmitted via Bluetooth, some observations can be lost during the transfer if the signal fails. Since this loss is generally random, we include a sensor as long as there are at least 69 days of data (~93% of days) and the missing days are not clumped at the end of the study period (indicating battery discharge, not random loss). Hence, our data cover six weeks before the lockdown started on March 27, seven weeks under full restrictions (Level 5), and two weeks where recreation was allowed between 6:00 am– 9:00 am (Level 4). The extended dataset with fewer sensors includes all of Level 4 (four weeks) and three weeks of Level 3, when most movement restrictions were lifted, but gathering restrictions were still in place.

To detect changes in nighttime motion over time, we first compare average activity across all weeks beginning on Feb. 14, 2020 and average activity across the different stages of lockdowns (Levels 5, 4, and 3). Moreover, to detect the drivers of changes in motion in the weeks during the lockdown in comparison to the weeks before, we compare average nighttime activity before and after March 27, 2020 for different days of the week and different times of the evening, night, and early morning by estimating Eq (1):

$${Activity}_{it}=\beta_{0}+\beta_{1}{LOCKDOWN}_{t}+\beta_{2}\theta_{t}+\beta_{3}({LOCKDOWN}_{t}\times \theta_{t})+\alpha_{i}+\varepsilon_{it}$$ (1)

Where *Activity*_*it*_ is the average five-minute motion or trigger count on path/compound *i* at time *t*, *θ*_*i*_ is a vector of dummy variables, one for each day of the week (or hour of the night for the second regression). *LOCKDOWN*_*t*_ is coded as 1 beginning at midnight on March 27, 2020 and afterward, and is coded as 0 before. We run all specifications with and without sensor fixed effects, where α_i_ refers to sensor fixed effects. *ε*_*it*_ is the robust standard error term. *β*_*1*_ indicates the change in five-minute motion after the onset of South Africa’s lockdown compared to average nighttime activity before. *β*_*2*_ is the average change in five-minute motion for day of the week (or hour of the night) compared to the constant. *β*_*3*_ shows the interaction effect of *LOCKDOWN*_*t*_ and the unit of time *θ*_*t*_ (day or the week or hour of the night). To check if there is a statistically significant difference between average activity during Level 5 compared to Level 4 and Level 4 compared to Level 3, we use a Welch’s Two-sample *t*-test of difference in means. All analysis is conducted using R Version 4.0.4.

After a first quantitative analysis, we conducted qualitative semi-structured interviews in October 2020 with four members of our local field team—all of whom are residents of this informal settlements—to ensure that we incorporated the knowledge and experience of those who experienced the lockdown in this context. We presented figures depicting the main findings to the team members and asked open-ended questions about their observations of life under lockdown with respect to the units of time we analyze. The initial study using the sensors was approved by the Ethics Commission of ETH Zurich (EK 2019-N-19) and an extended approval for this study was granted in August 2020. The local leadership committee provided oral consent for the sensor project to take place in this area and written consent was obtained from each household on whose house or property a sensor was installed. Raw data contain no personally identifying information.

## Results

### Impact of lockdown on mobility

Over the entire study period from February 14 –May 14, 2020, the average five-minute motion between 6:00 pm– 8:00 am was 1.25 triggers per five-minute period on paths (*sd*: 2.72) and 1.02 triggers per five-minute period in compounds (*sd*: 3.2)—about 15 per hour in paths and 12.2 per hour in compounds. We analyze path and compound sensors separately, since they measure different types of activity. Sensors on paths generally measure people in transit, while sensors installed in compounds measure the activity of a few people in a small space.

**Fig 1:**
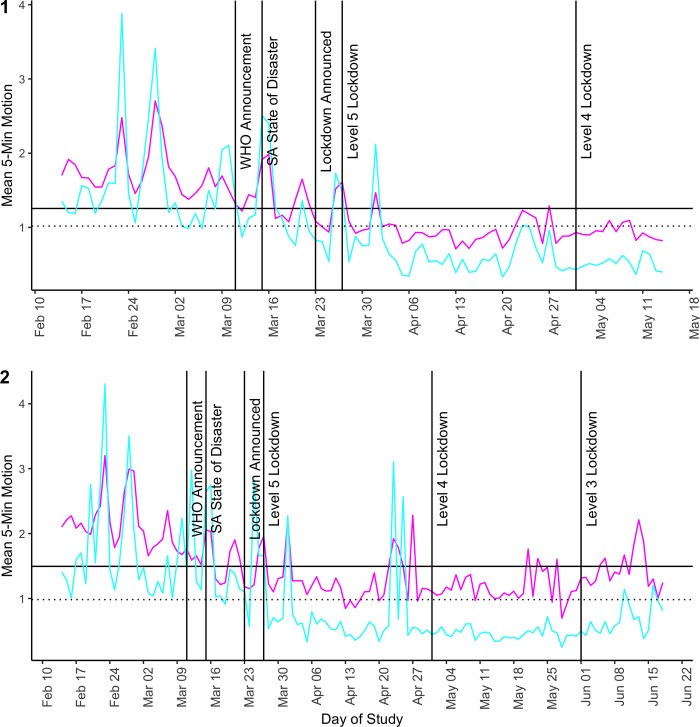
Average five-minute motion over the study period. The figures plot the average five-minute motion for each day of the study (Panel 1: Feb 14 –May 14; Panel 2: Feb 14 –June 18). The x-axis shows the date on every Monday. The pink line represents data from sensors installed in paths (Panel 1: 60; Panel 2: 21), while the blue line represents data from sensors installed in compounds (Panel 1: 26; Panel 2: 18). The black horizontal line is overall five-minute path average and the dotted black line is the overall five-minute compound average.

From pure visual inspection of Fig 1, which tracks the daily five-minute mean throughout the study, we see a steady decline in motion already at the beginning of March (before the national lockdown). Notably, that decline bottoms out and flattens shortly after South Africa implements an official lockdown on March 27, 2020. Moreover, the activity peaks associated with weekends visible in February, and to a slightly lesser extent in March, largely disappear in April (Level 5) and May (Level 4). Although some restrictions on morning activity were lifted in Level 4 and more people could potentially work, activity remains low. With Level 3, activity in June (Fig 1, Panel 1) rises again, consistent with the loosening of many restrictions, but mobility remained lower than in February before the first COVID-19 cases occurred. In Fig 1, the bottom panel shows means from sensors that worked until Level 3. Although the pattern is less smooth, due to greater variance, it is very similar to the top panel, where more sensors are included.

**Fig 2:**
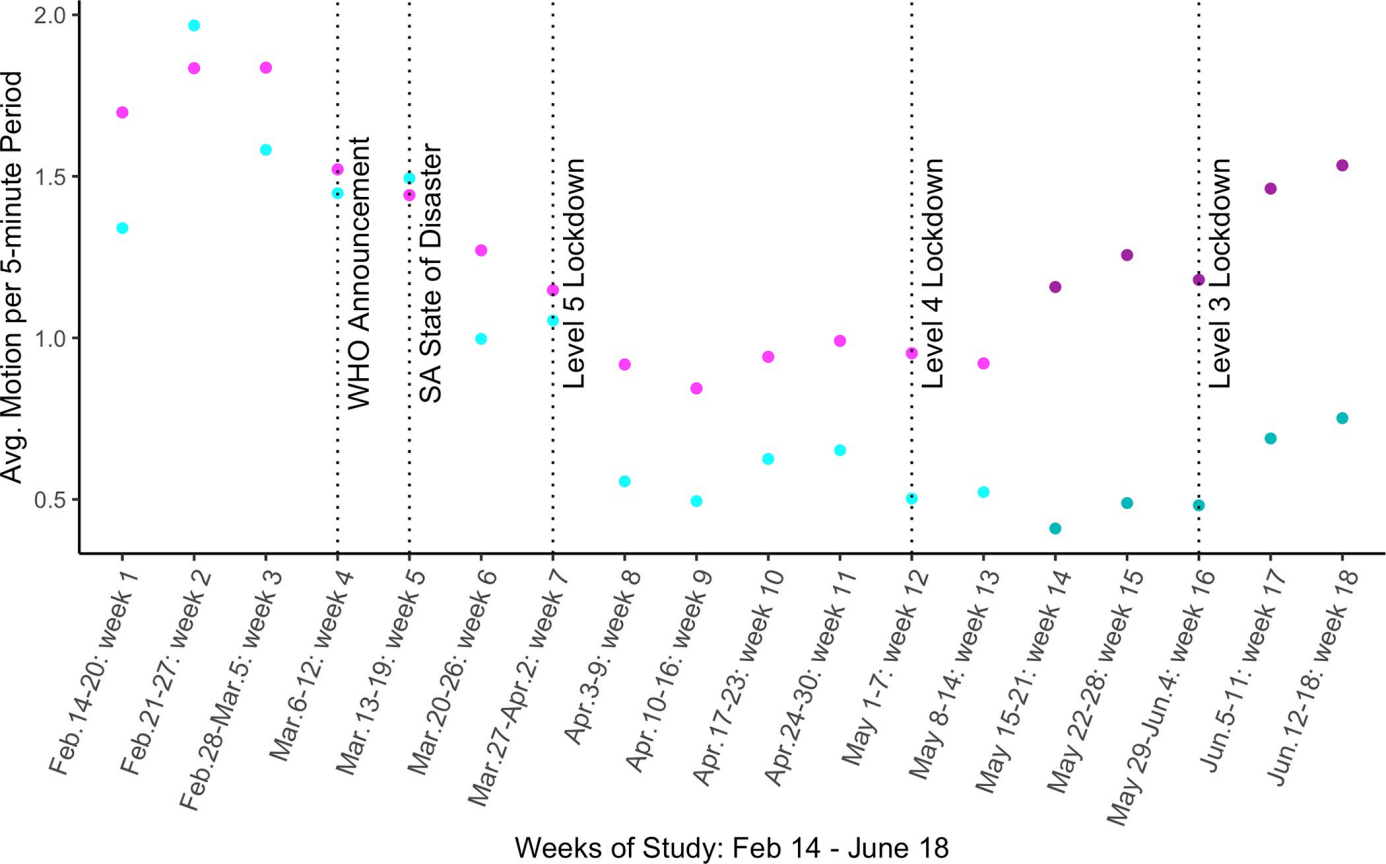
Average five-minute motion by week of the study. The plot shows mean five-minute motion by week of the study. Pink points represent path means; blue points represent compound means. Underlying data is the same as in Fig 1. Dark pink and dark blue points indicate means from the restricted dataset that adds weeks 14–18 to the study period. Means in the restricted dataset prior to week 14 are generally similar, but the pattern is a bit noisier. The coefficient is significant in each week in the main study period (weeks 1–13) for both paths and compounds. See S1 Table. A version of this graph with only data from the restricted dataset see S4 Fig.

If we look at week-to-week changes in average nighttime activity (Fig 2), the change in average motion is significant throughout the study period. We find that in the paths (pink dots), nighttime motion steadily and significantly declines between February 28 and March 26 (23% in weeks 4–6, Fig 2, S1 Table)—the three weeks before the lockdown. Compound nighttime motion (blue dots) is more erratic in the month prior to lockdown. Still, an overall decline (19% in weeks 4–6) in March is evident (Fig 2, S1 Table).

In week 7, the first week of lockdown, the decline in average five-minute motion in paths seems to follow the pattern, with a similar decline in mean motion between week 6 and week 7, but in the second week of lockdown (week 8: Apr. 3–9) average five-minute motion drops sharply below one trigger per five-minute period. Weeks 9–11 (Level 5) remain relatively stable (S1 Table, column 1). Also, in the first two weeks of Level 4 (weeks 12 and 13), when more outdoor activities were allowed again, outdoor mobility remained low.

To see how nighttime activity evolved over the extended study timeline, we show average weekly five-minute motion in Fig 2 for weeks 14–18 from the smaller sample of sensors. By weeks 17 and 18, the second and third week of Level 3, we already see nighttime activity levels in paths have returned to levels similar to weeks 5–7 (just before and at the start of Level 5). If we compare the means for weeks 16–18 from the extended data to the means for weeks 5–7 from the main data, the means are practically similar enough to indicate a return to pre-lockdown nighttime activity (S1 Table, column 3), but not to “normal” outdoor activity (weeks 1–3).

Compound nighttime motion follows a similar pattern to paths, however, activity dropped more slowly in compounds than in paths before the lockdown, but then more drastically once lockdown was announced. Moreover, nighttime activity also seems to rebound for compounds when Level 3 was announced, but does not—in contrast to paths—come close to pre-lockdown levels. Again, comparing weeks 5–7 from the main dataset to weeks 16–18 from the extended dataset, respectively, the five-minute means in June are only half as large as the three weeks prior to lockdown in March.

When we compare mean five-minute motion during February 2020 to mean motion during lockdown (March 27 –May 14, 2020), we find that five-minute motion decreased by 48% in paths. Compared with the entire pre-lockdown period in our study (February 14 –March 26, 2020), on average, the lockdown is associated with a 40% decrease in path pedestrian triggers per five-minute period during the lockdown (p < 0.01), bringing the mean number of pedestrians from 1.6 per five-minute period to 0.96 per five-minute period. In other words, on average, prior to the lockdown sensors measured about 19 triggers per hour, while after lockdown the total was about 11.5 triggers per hour (S2 Table, column 1). Then, using results from S1 Table (column 1), we can separate out the effect of activity declines in March, when awareness of COVID-19 was growing, and the effect of lockdown. Nearly 50% of the decrease in activity after February can be attributed to reduced activity in March, and the remaining to the lockdown.

In compounds, the decrease in nighttime compound activity under lockdown is larger. Comparing the five-minute mean during lockdown to February 2020, there is a 61% decrease in motion. Compared to the entire pre-lockdown period, the lockdown is associated with a 57% decrease in nighttime triggers during lockdown (p < 0.01), with an average of 0.63 triggers per five-minute period, which is about 7.56 triggers per hour, down from about 17.65 triggers per hour, on average, before lockdown (S2 Table, column 9). Again, using the results from S1 Table (column 2), we see a 19% reduction in activity between February (weeks 1–3) and the three weeks before lockdown (4–6), which captures about 32% of the overall decrease in nighttime activity after February—thus, COVID-19 awareness appears to have a smaller effect in these small, semi-private spaces compared to paths.

If we change the lockdown date to March 11 (the WHO pandemic announcement) or March 15 (South Africa state of disaster announcement) the results change very little (results available from the authors upon request). We also re-run the analysis controlling for each lockdown level (Levels 5, 4, and 3) separately using both the larger data set with 60 path and 26 compound sensors (but only up to May 14, 2020) and the smaller data set with 21 path and 18 compound sensors (up to June 18, 2020) and find that Level 5 and Level 4 show very similar activity patterns. Only in Level 3 do activity patterns resume on paths, but not so in compounds (S2 Table, columns 5–8 and 13–16).

### Impact of government regulations on activity

**Fig 3:**
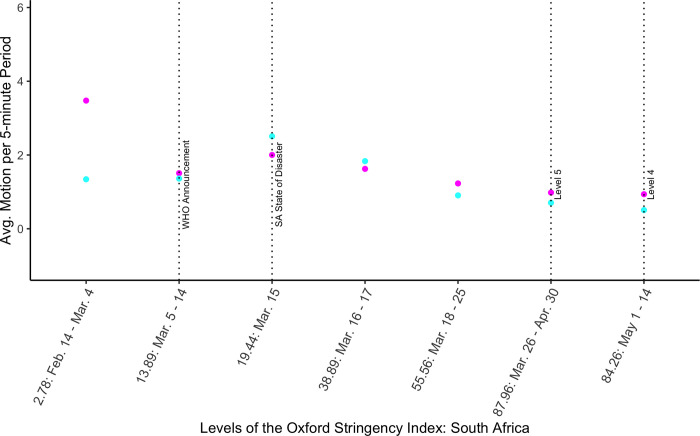
Average 5-minute motion by levels of the Oxford Stringency Index for South Africa. Pink dots represent path averages and blue dots represent compound averages.

The previous analysis suggests that the implementation of lockdown restrictions in South Africa and any coincident increase in awareness of COVID-19 was fuzzy. To check this hypothesis, we use Oxford University’s Coronavirus Government Response Stringency Index (SI) for South Africa, which also tracks government COVID-19 measures by day for many other countries. The index value does not necessarily change daily, so we replace the lockdown dummy with the SI index coded as a categorical variable. We see that activity is not simply decreasing in lock-step with increasing levels of stringency. Fig 3 (columns 2 and 4 in S3 Table) shows how mean motion changes with each of the seven SI levels during the study period. All changes are significant at the 99% level and are calculated with respect to the first level (2.78). For both paths and compounds, the level 19.44 (mid-March, week 5), the day a state of disaster is announced, is associated with a significant increase in nighttime motion, but it is only one day. For compounds only, level 38.89 (also week 5) is also associated with a significant increase in activity. Fig 3 reflects the results discussed above: the largest decrease in motion actually seems to occur between levels 38.89 and 55.56 (weeks 5 and 6), the week the state of disaster is announced and the week the lockdown is announced, but the lowest average motion is still during the lockdown (87.96 in Level 5 and 84.26 in Level 4).

### Impact of lockdown on daily and hourly mobility

To analyze what is driving the significant drop in average nighttime activity in both paths and compounds, we study the effect of the lockdown on particular days of the week and hours of a day (see Eq 1 and S4 and S5 Tables).

**Fig 4:**
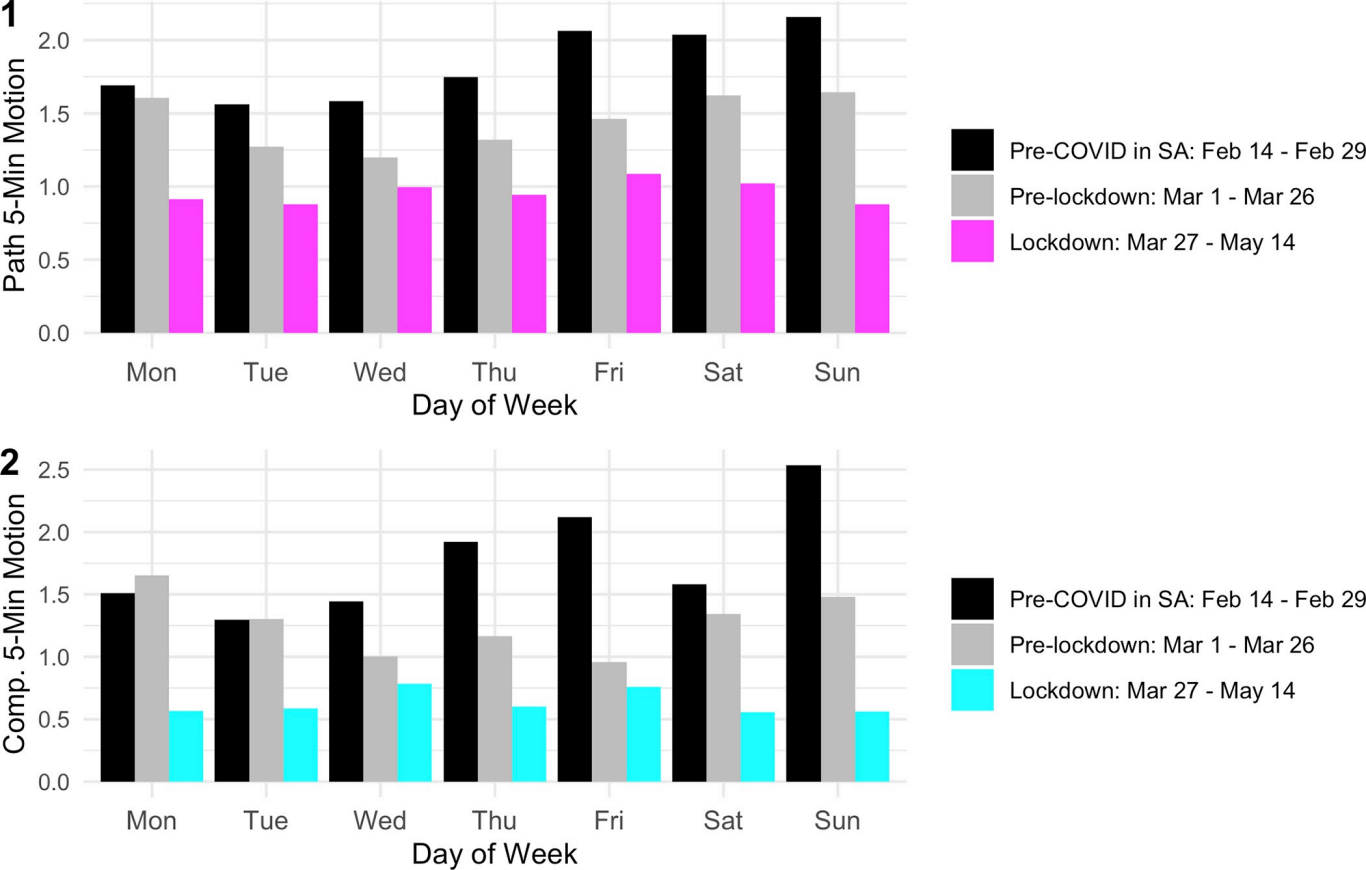
Average five-minute motion by day of week. The graphs show the five-minute average by day of week for the month of February, the month of March before lockdown, and for the lockdown period. Table of results with February included separately available from the authors upon request.

In both paths and compounds, the largest decreases in average five-minute motion are seen on Saturday and Sunday nights (a decrease of 44% and 52% in paths, respectively, and 62% and 69% in compounds, respectively; S4 Table). Prior to the lockdown, the average week follows a cycle with higher nighttime activity during the weekends. During the lockdown the cyclical pattern disappears and all of the nights look remarkably similar (Fig 4), indicating that residents were mostly restraining outdoor social activities (on the weekend) and, to some extent, non-essential travel during the week. If we interact each lockdown stage with each day of the week, we see little difference between Level 4 and Level 5 in both paths and compounds (results available from the authors upon request).

**Fig 5:**
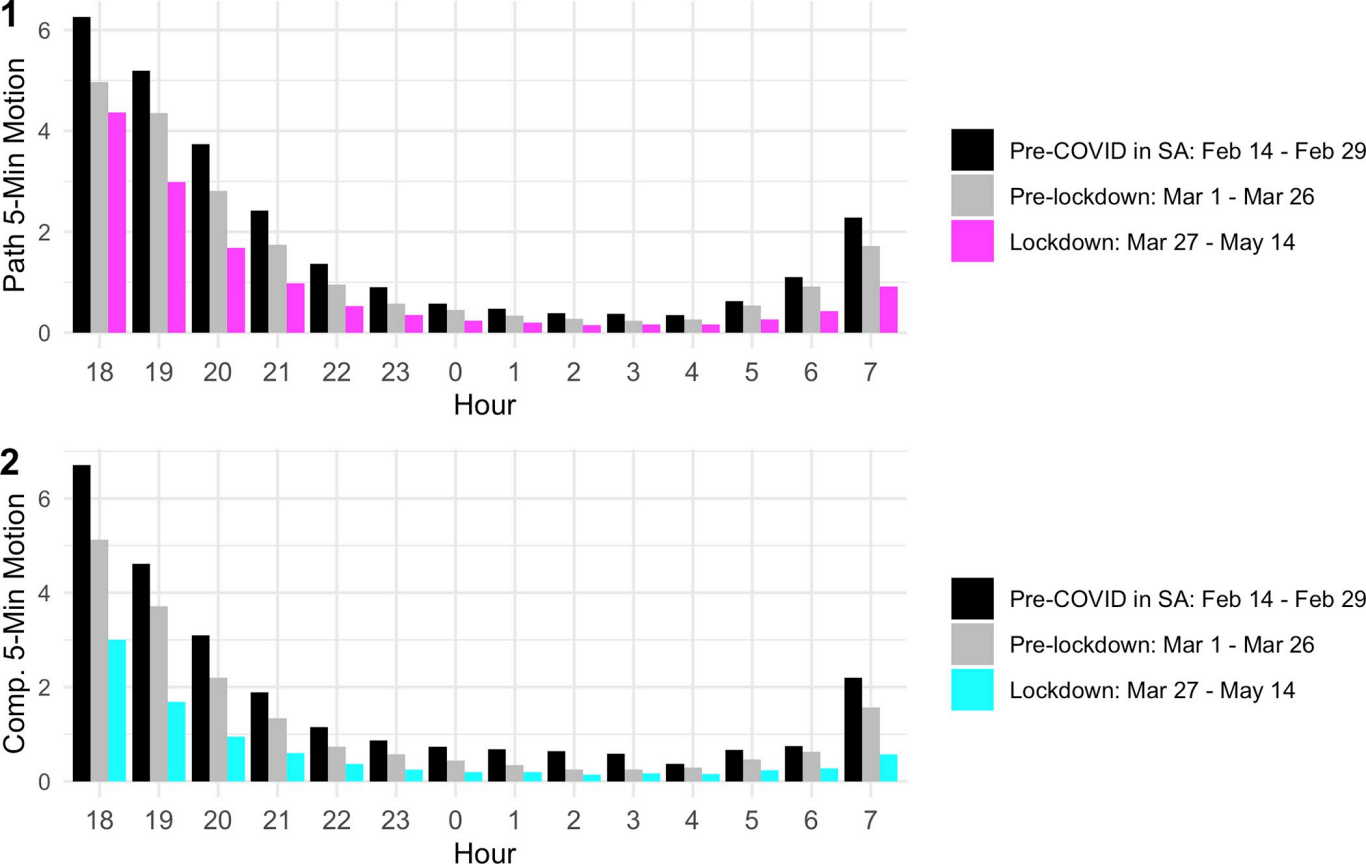
Average five-minute motion by hour. The panels show the average five-minute motion by hour in the dataset for the month of February, the month of March before lockdown, and for the lockdown period for paths (1) and compounds (2), respectively. Table of results with February included separately available from the authors upon request.

In a next step, we study the hours that drive activity declines. In paths (Fig 5, Panel 1, S5 Table, columns 1 and 2), there is a significant decrease in activity during lockdown for every hour (p <0.01 for all hours except hour 5; hour 5 is p < 0.1). The largest decreases (in absolute terms) are between 6:00–9:00 pm, and from 6:00–8:00 am. The results indicate that people are curtailing activity around the primary commuting and social times, but the reduction, especially between 6:00–9:00 pm, is not as large as expected given that it was against the law to be outside for non-essential reasons under the lockdown during these times.

In compounds (Fig 5, Panel 2), the largest decreases (in absolute terms) are also between 6:00–9:00 pm, as well as from 7:00–8:00 am (S5 Table, columns 3 and 4). In both paths and compounds, the small absolute differences between means before and during lockdown in the middle of the night might indicate that motion then can be attributed to activities that residents cannot avoid, e.g., going to the toilet, etc., unlike social activities, which can be avoided in the evening hours.

One key difference between Level 5 and Level 4 of the lockdown is that South Africans were allowed to be outdoors for recreational (non-essential) activity between 6:00 and 9:00 am beginning May 1, 2020. Despite this rule relaxation, when we interact lockdown level with hours of the day, we do not see a significant increase in path activity between 6:00–8:00 am from Level 5 to Level 4 (results are available from the authors upon request). These results suggest that although there were slight changes to the rules between Level 5 and Level 4, residents did not radically change their nighttime behavior, at least not in early May.

## Discussion

Despite concerns about lockdown compliance in informal settlements, we find significant reductions in activity between 6:00 pm and 8:00 am on pedestrian paths (down by about 48%) and in shared, semi-private spaces called compounds (down by about 61%) compared to activity in February 2020. Importantly, activity already started to decline three weeks before the lockdown, particularly in paths (already 23% in March), when COVID-19 was quickly spreading worldwide and South Africa declared a state of emergency. The results are similar when we use the Oxford Stringency Index as the explanatory variable.

However, motion in the evening, nights, and early mornings never disappeared during lockdown, even when it was against the government rules. Our results further show that after the lockdown, each day began to look similar with regard to motion, rather than following the usual ebb and flow of a typical week, which tends to have higher weekend activity in residential areas. In other words, activity decreased the most during weekends. Moreover, we find that in evenings and mornings—typical commute hours—activity decreased more than during the late evenings and nights. Although we see the largest reduction during commute hours, we still see more activity in those hours, on average, than in the middle of the night.

Taken together, the reduction in nighttime activity in this informal settlement indicates more compliance with the lockdown regulations than was portrayed in many media reports, but also that people reduced activity in response to growing awareness of COVID-19 before lockdown. This finding is consistent with other studies [26, 28], in particular a study in the US by Cronin & Evans [26], who find dramatic mobility declines between March 8–14, 2020 prior to the onset of most lockdowns, but when many areas had announced a state of emergency. They find that state of emergency declarations account for 7–28% of the declines they measure. When we compare week-to-week five-minute motion means in March to February, we see that the decline for paths is on par with their results (23%). For compounds, however, the week-to-week change in March is inconsistent, but overall there is still a 19% decrease compared to the three first three weeks of the study. This difference in the nature of activity that occurs in paths compared with compounds suggests that behavior linked to transit, rather than say, socializing outdoors, may have been just as influenced by growing media attention to COVID-19 as the lockdown, while the lockdown may have been more influential in driving activity reductions in compounds.

Moreover, in the first week of lockdown the five-minute mean (1.15) is not as low as in subsequent weeks. One explanation is that residents had not realized how strict the implementation would be or how serious the threat was and therefore, had not yet dramatically adjusted their behavior. When we showed the results to our local field workers, they had two explanations. First, the first day of lockdown was the last Friday of the month, when many workers receive wages. In the absence of COVID-19, they would expect March 27 to have more nighttime activity than the previous weeks because most people would have just received a paycheck. Payday, in concert with pressure to prepare for lockdown, may have motivated a flurry of activity. Second, they said that while many people were fearful of the virus, others did not take lockdown regulations seriously until they saw on TV that other countries also had lockdowns and until the police and army began enforcing restrictions. Durizzo et al.’s [8] results echo this speculation—they find that the South Africans in their sample are more likely to perceive the government’s actions against COVID-19 as too extreme and tend to underestimate the number of cases in the country. In addition, in comparison to Ghanaians, South African respondents tend to the extremes—either they followed most or none of the rules. They also find that more than 80% report informing themselves about the pandemic by watching TV.

**Fig 6:**
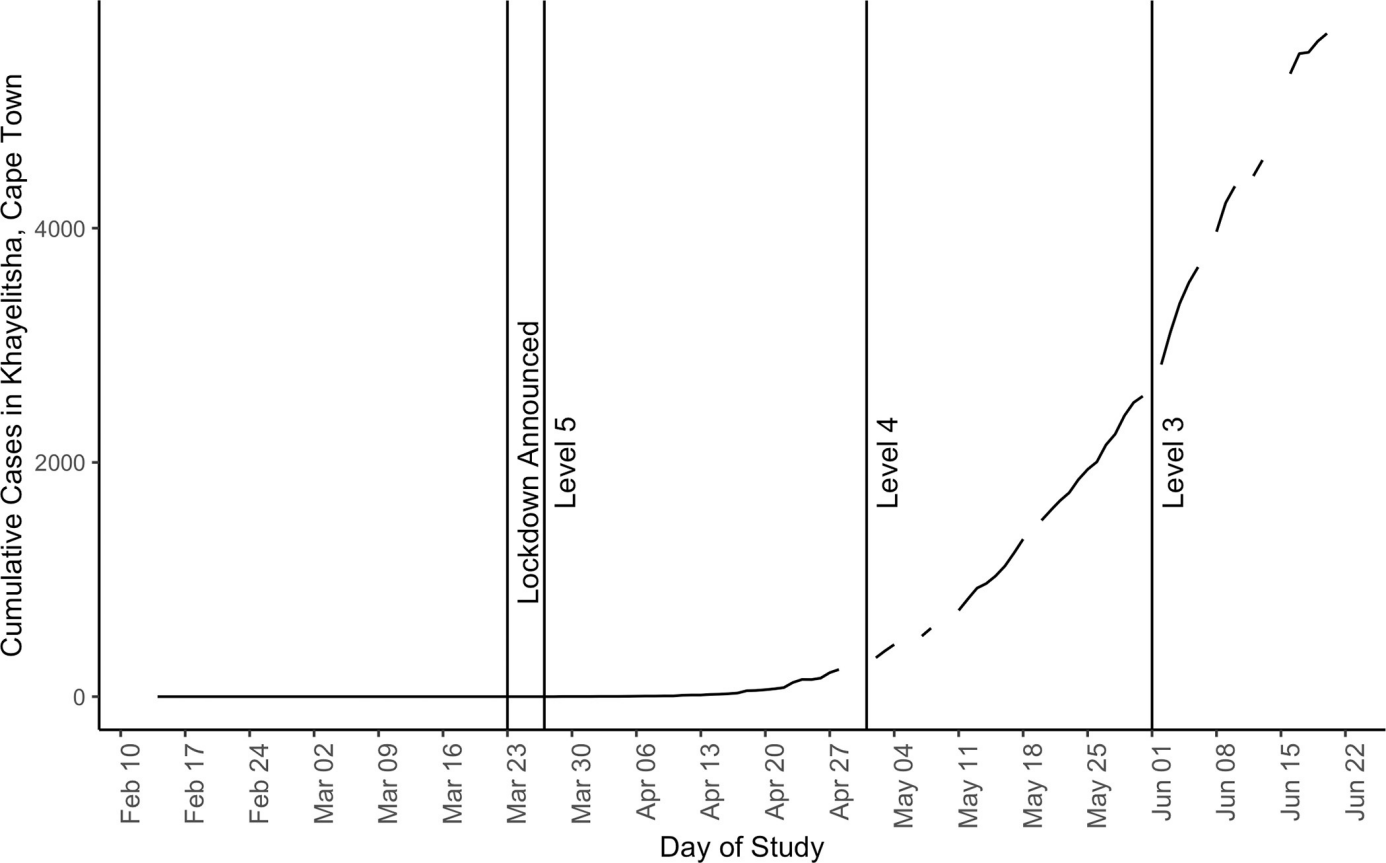
Cumulative reported cases of COVID-19 in Khayelitsha, Cape Town. These data were collected by the authors from press releases put out by the Western Cape Premier Alan Winde. Gaps in the line indicate days in which no press release with Khayelitsha-specific data was publicly available.

Furthermore, the first COVID-19 case in Khayelitsha was not documented until March 29, 2020, supporting the point that the risk may not have been salient right away. Fig 6 shows the COVID-19 case trajectory in Khayelitsha from February 14 –June 20, 2020. Indeed, there were few reported cases until Level 4 lockdown began in May 2020. The field team members said they knew of only one person from the neighborhood who had tested positive for COVID-19 as of October 2020. While other research indicates that at least some mobility restrictions limit the spread of COVID-19 to some extent [49, 50], due to the limitations of our data we cannot say anything about how the changes in nighttime activity we observe relate to the spread of COVID-19, however, the case data contextualize what was going on in the vicinity of the informal settlement then.

Activity declines in paths of 48% and in compounds of 61% (compared to February), while generally lower than those found in higher-income countries [36, 37], are consistent with findings based on Google Mobility data in several African countries [9], Google Mobility from the Western Cape, the province where this informal settlement is located, which includes formal and informal areas (S3 Fig), and a study using phone location data in Mumbai [19]. In comparison, two Swiss mobility studies find activity drops more sharply at the start of the lockdown (though they decline somewhat beforehand), but mobility levels climb more quickly afterwards [36, 37]. Though, notably, the lockdown in South Africa was much stricter than in Switzerland. One reason we might observe such a substantial drop in activity is that residents did not socialize outside after dark. Another reason could be that if residents commute less other activities that might occur at night, such as doing evening chores and procuring food, may instead happen during the day.

While we do not know which activities drive our results, the day-of-week and hour-of-day analyses provide ideas. The day-of-week analysis shows large declines in weekend activity (S4 Table). Our local field team said they observed fewer people out on weekends, but some may have just socialized indoors. The hour-of-day analysis shows that activity does not disappear, even at specific times when it is not allowed (e.g., after 8:00 pm during Level 4). Our team suggested that declines in the evening were not as large as the morning (S4 Table) because there were always some people out, though fewer than normal. From around midnight to 4:00 am, the field workers attribute the decrease in activity to fewer criminals out in paths. This interpretation tracks with reports that crime was markedly down early in the lockdown [51, 52].

Strangely, we notice that although people were allowed to be out for recreational activities between 6:00 am and 9:00 am under Level 4 restrictions, we observe a decrease in activity between 6:00 am and 8:00 am. Our local field staff said people may not have taken advantage of the rule relaxation because it was dark until around 7:20 am (sunrise) and that those who did exercise left the informal settlement, so they likely produced few additional triggers. Notably, June in the southern hemisphere is analogous to December in the north, meaning these are some of the longest nights of the year in Cape Town.

Larger activity reductions on weekends and in compounds (in comparison to paths) suggest that the nighttime activity that persisted after lockdown was mainly driven by pedestrian activity, rather than more stationary activities like socializing, which may explain the large decline in compound activity once the lockdown was in place, but not before. The time patterns we observe indicate that, even when it is against regulations, residents may go out to secure basic necessities, use sanitation infrastructure, socialize despite restrictions, or pursue economic opportunities even if they risk COVID-19 exposure.

### Robustness checks and limitations

Part of the effect we document could be explained by seasonality and/or temperature. March is the end of summer/early fall in Cape Town, so days are getting shorter and cooler throughout the study. On Feb. 14, 2020, sunrise occurred at 6:19 am and sunset at 7:40 pm, while on May 14 sunrise was only at 7:31 am and sunset as early as 5:53 pm [53], so the daytime was about three hours shorter. To see if seasonality drives our results, we drop observations between 6:00 and 7:00 pm and between 7:00 and 8:00 am, since these hours were sometimes, but not always dark during our study period, and then re-run the main analysis. We find a 45.8% decrease in paths and a 59.9% decrease in compounds, a similar effect as in our main results, suggesting seasonality is not a main driver (S6 Table).

Moreover, using the full range of hours as well as hourly weather data for Khayelitsha from OpenWeather Map, we re-run the main analysis controlling for hourly temperature, as well as time effects (hour of day and day of week dummies) [54]. In paths, we find lockdown is associated with 6.6 fewer triggers per hour (about 0.5 per five-minute period), which is a smaller decrease than in our main result (7.7 per hour or 0.64 triggers per five-minute period), but significant at the 99% level. In compounds, lockdown is associated with 9.6 fewer triggers per hour (about 0.8 per five-minute period), which is just a slightly smaller decrease (also significant at the 99% level) than in the main results (10 per hour or 0.84 triggers per five-minute period) (S6 Table). We conclude that temperature does have some mitigating effect on the relationship between lockdown and nighttime activity, as expected, however, this effect does not substantially alter the conclusion based on our main results.

Although our main results appear to be robust, there are several limitations that should be considered when assessing these findings. First, since we do not collect accurate data for all 24 hours of the day, we cannot study how daytime activity changed in response to lockdown and whether nighttime activity is displaced to daytime hours (which can be seen in Google Mobility data for the Western Cape in S3 Fig). While this limitation is not a substantial problem in the analysis for which the sensors were intended, it leaves us with a gap in understanding the response to lockdown in informal settlements. Second, due to sensor attrition we do not have as much data for all of Level 4 (May 2020). Still, as our analysis using the smaller dataset shows, this limitation may not have a major impact on our main result. In addition to study duration, sensor attrition also limits the overall sample size in an already somewhat small informal settlement. Out of 121 path sensors and 50 compound sensors originally installed, only about half have complete data in the relevant time period. Even though we do not believe there is systematic bias in attrition, as we do not observe clustering of dysfunctional sensors, we cannot entirely rule it out. Furthermore, sensor attrition prevents us from knowing whether our estimates are upper or lower bounds.

The third limitation has to do with the nature of the sensor data. Since we know nothing about the passersby, it is difficult to be certain if the count represents several unique individuals or one person repeatedly triggering the sensor. This uncertainty is particularly problematic in compounds. Since compounds do not allow through traffic and frequently have a gate that is locked at night, any activity detected by the sensors is probably from the residents sharing the compound, rather than someone in transit. Just one person in a compound can create many triggers just by moving a lot within the space, therefore, less activity by even one person could potentially create an outsized effect on the trigger counts recorded by the sensor.

Although mobile phone data does not suffer from this limitation or limits on daytime measurement, they have other shortcomings: in informal settlements mobile phones are often shared [19], in our context pre-paid cellular data is expensive and not always affordable, and mobility tracking apps are limited by GPS accuracy, making it hard to identify tracks. Furthermore, in our setting we learned from an earlier survey that only 38% of respondents report carrying a mobile phone outside with them at night for fear of theft. Thus, using mobile phone data would likely have created larger measurement errors.

## Conclusion

Despite both widespread concerns about lockdowns in informal settlements and highly publicized skepticism about whether residents in these neighborhoods adhere to them, we find quantitative evidence that residents in one informal settlement in Cape Town, South Africa significantly limit nighttime mobility in response to state-mandated lockdowns of public life, but also in response to media coverage of and government communication about the pandemic prior to the lockdown. Using nighttime motion sensor data from a pre-existing project, we show activity began declining throughout March by 23% in paths and 19% in semi-private shared spaces, called compounds (in comparison to February) when COVID-19 cases were on the rise in Europe and the US, but that the lockdown itself had a substantial additional and sustained effect. Evening, nighttime, and early morning motion in paths went down by 48%, in comparison to February. In compounds, activity decreased by nearly 61% in comparison to February. Breaking this result down by day of week and hour of day, we find the largest decreases in nighttime activity on Saturday and Sunday and during commute hours between 6:00–9:00 pm and 6:00–8:00 am. These findings are consistent with the regulations in place in South Africa at the time—that is, a ban on all non-essential social activity (including alcohol and cigarettes) and a sharp reduction in businesses allowed to operate, resulting in severe unemployment [16].

The motion sensors we use to gather data for this analysis only record accurate date in the evening, night, and early morning and record no details about passersby, meaning the data are helpful for understanding the use of public space at night, but not for learning more about factors that might be driving compliance and non-compliance, i.e., *who* is using it or *why*. In addition, although we use hyper-local data in an under-studied context (also as a result of lockdown regulations in place worldwide) and we cannot identify causal effects, our results are remarkably similar in direction and magnitude to mobility studies of developing countries using much larger datasets, like Google Mobility data [9, 19, 34, 35, 55].

Accounting for competing factors, such as weather and daytime seasonality, does not change our results much. Therefore, changes in behavior due to less daylight do not seem to drive the results. When we control for temperature, the activity reduction in both paths and compounds is somewhat smaller, but still significant. Therefore, it seems plausible that people were not just following the law or staying inside more due to bad weather, but also reducing activity because a) they were aware the virus is dangerous and b) there may have already been less work or fewer social events prior to the official lockdown since recommendations to social distance and school closures began nearly two weeks prior.

While we may demonstrate descriptive evidence that compliance with social distancing measures is possible in informal settlements to some extent, that does not necessarily mean that broad lockdowns are the most effective strategy in these neighborhoods. Although evening and early morning activity in both paths and compounds was significantly lower, it never entirely disappeared, suggesting that either a) people were trying their best to constrain their time in public, but certain activities were either essential, too important for other reasons to give up, or not considered to be dangerous or b) residents had displaced as much outdoor activity to daylight hours as possible (which we cannot accurately measure) and what we measure is a level of nighttime activity that is unavoidable in an informal settlement. Furthermore, activity was already declining in paths prior to the lockdown, suggesting that information and less strict measures also had an influence.

Still, it is notable that activity reaches a low point shortly after the start of lockdown and stays low through the end of the main study period, in contrast to the Swiss examples using phone tracking apps [36, 37] where residents increase mobility again shortly after lockdowns are put in place. While the Swiss lockdown was far less stringent than in South Africa (see S2 Fig), the comparison illustrates why sensationalized media reports that residents of informal settlements did not follow lockdown rules are likely misrepresentations. The longer lasting declines we observe may also result from a combination of compliance with regulations as well as economic devastation, if the declines are atleast partially linked to residents being unable to return to the workforce, as was documented by Gil et al. in informal settlements in Chile [12].

Without knowing the precise mechanism motivating residents’ choices about when and how much to adhere to lockdown regulations, it is still possible to discuss potential lessons for policy. The fact that activity during nighttime hours, especially commute hours, decreased, but still represented the highest level of activity throughout the measured hours indicates that lockdown regulations reduce outdoor activity, but that future efforts to manage contagious diseases must focus on the activities taking place during these time periods. First, mitigating the need for people to leave their homes to access sanitation or water could lead to further reductions in activity and reduce the number of contact points with neighbors. Improving access to sanitation or water need not merely be seen as a response to a pandemic, but could be incorporated into longer term plans to upgrade informal settlements, by prioritize solutions that take public health emergencies into account. Second, pandemic response should not ignore the sheer density of informal settlements and the fact that households tend to be multigenerational and fluid. Since homes are small, many otherwise private activities take place in shared or semi-private spaces, such as washing or hanging laundry. Rather than viewing these activities as so-called non-compliance, public health officials should account for these needs in designing lockdown measures for informal settlements. If residents perceive social distancing restrictions have been designed with the realities of their daily lives in mind, they may be even more willing to comply. Third, addressing the fact that most residents of informal settlements cannot sustain long periods without work and often need to work outside the home is essential. It is impossible to ask people to forego the basic activities and needs of daily life. Therefore, policymakers cannot implement a single, broad-spectrum lockdown policy, but rather should consider what compliance means in different areas, such as the unique characteristics of life in informal settlements.

## Supporting information

S1 Fig Pedestrian motion sensor. A pedestrian motion installed in an informal settlement in Cape Town, South Africa.
(TIF)

S2 Fig Oxford coronavirus government response stringency index for five countries. Using the Oxford Coronavirus Government Response Stringency Index, the figure shows the evolution of government responses to COVID-19 in South Africa (ZAF), Ghana (GHA), Kenya (KEN), the United States (USA), and Switzerland (CHE) over the course of the study period.
(TIF)

S3 Fig Google mobility data for the Western Cape, South Africa from February 15 –May 14, 2020. Data downloaded from Google COVID-19 Community Mobility Reports. The Western Cape is the province which encompasses Khayelitsha and the City of Cape Town. Percent changes in activity are calculated with reference to Jan 3 –Feb 6, 2020.
(TIF)

S4 Fig Average five-minute motion by week. The plot shows mean five-minute motion for every week of the extended study until June 18, 2020 with the reduced sample of sensors. Pink points represent path means; blue points represent compound means. See S1 Table for regression results.
(TIF)

S1 Table Average five-minute motion by week of the study. (PDF)

S2 Table Effect of South Africa’s lockdown on nighttime activity. (PDF)

S3 Table Results of OLS regression using the Oxford Stringency Index (SI) as the predictor. (PDF)

S4 Table Effect of lockdown by day of week. (PDF)

S5 Table Effect of lockdown by hour of the day. (PDF)

S6 Table Robustness checks. (PDF)
