# Supplementary figures and images for: Mobility in informal settlements during a public lockdown: A case study in South Africa
Source: PLoS One. 2022 Dec 22;17(12):e0277465. doi: 10.1371/journal.pone.0277465 (PMC9778567; doi:10.1371/journal.pone.0277465)

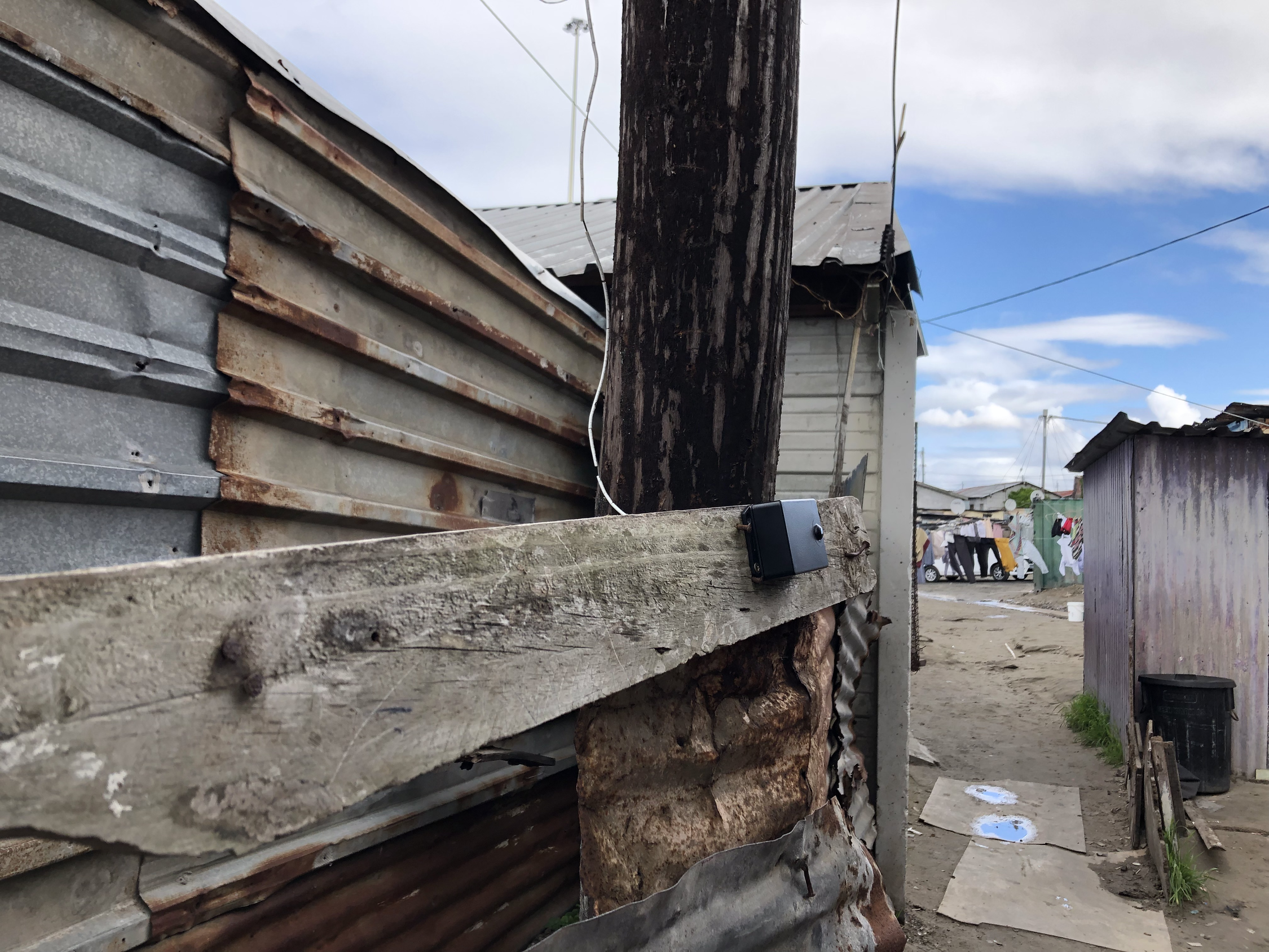

Supplement: S1 Fig — A pedestrian motion installed in an informal settlement in Cape Town, South Africa. (TIF) [file pone.0277465.s001.tif]

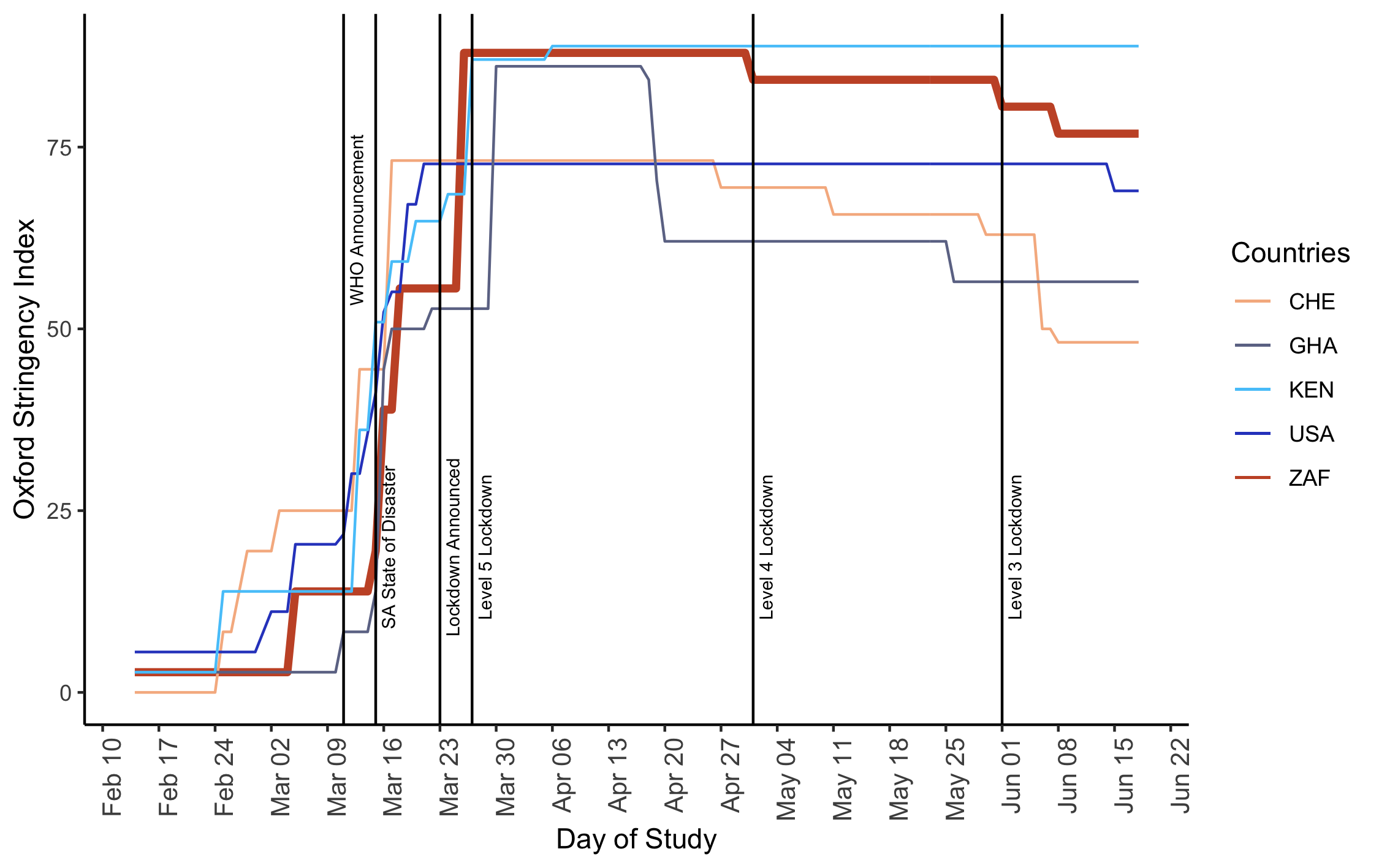

Supplement: S2 Fig — Using the Oxford Coronavirus Government Response Stringency Index, the figure shows the evolution of government responses to COVID-19 in South Africa (ZAF), Ghana (GHA), Kenya (KEN), the United States (USA), and Switzerland (CHE) over the course of the study period. (TIF) [file pone.0277465.s002.tif]

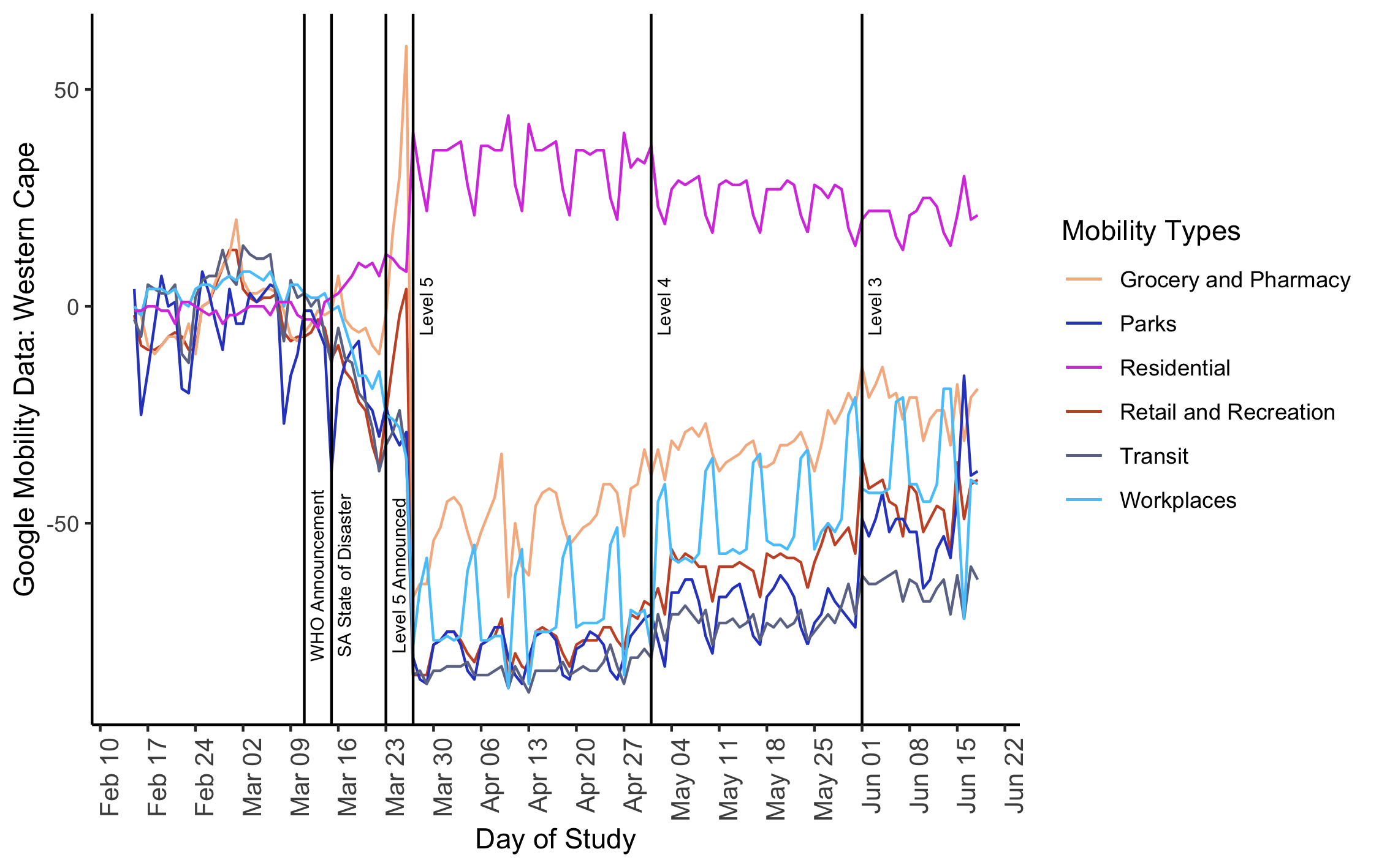

Supplement: S3 Fig — Data downloaded from Google COVID-19 Community Mobility Reports. The Western Cape is the province which encompasses Khayelitsha and the City of Cape Town. Percent changes in activity are calculated with reference to Jan 3 –Feb 6, 2020. (TIF) [file pone.0277465.s003.tif]

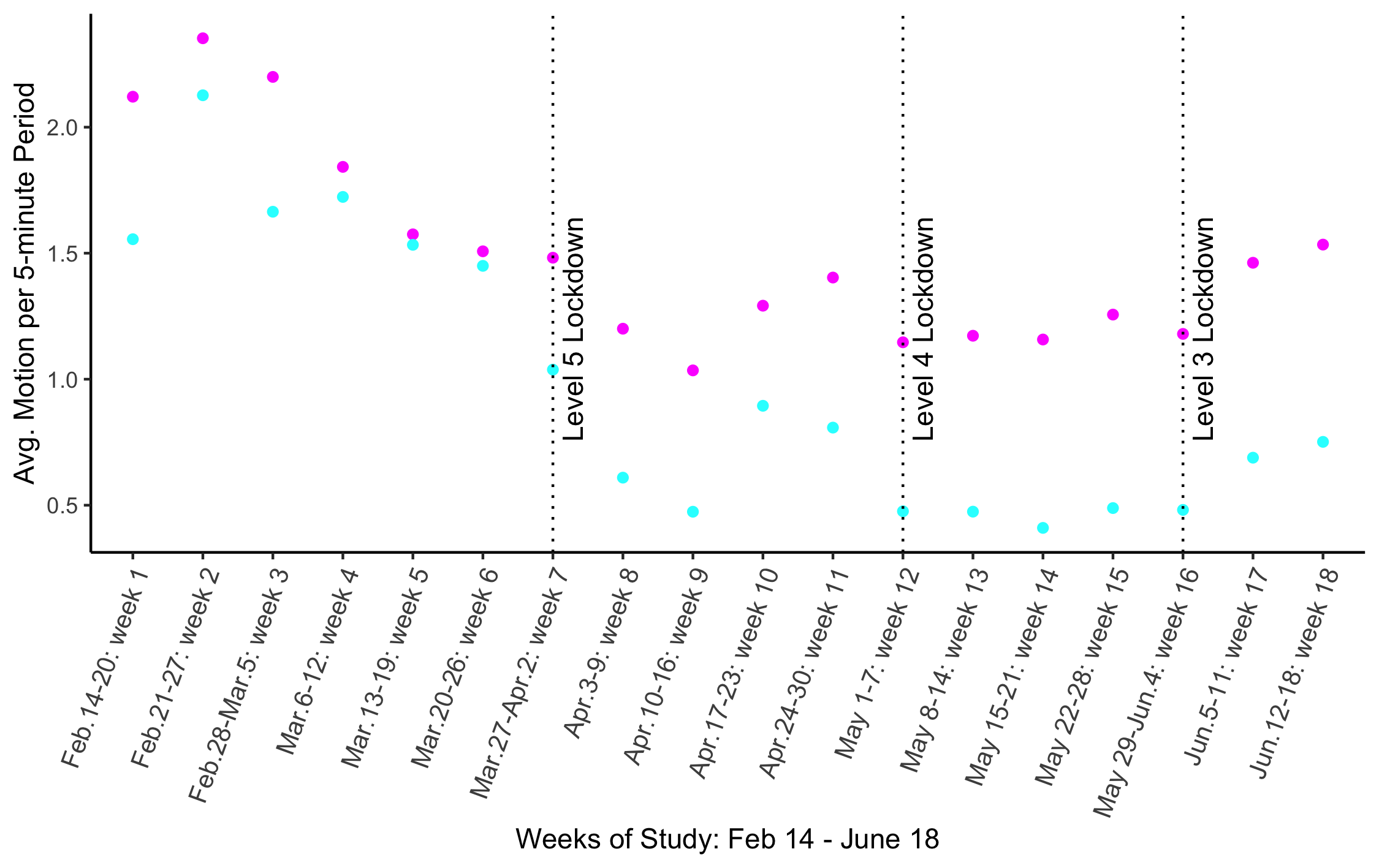

Supplement: S4 Fig — The plot shows mean five-minute motion for every week of the extended study until June 18, 2020 with the reduced sample of sensors. Pink points represent path means; blue points represent compound means. See S1 Table for regression results. (TIF) [file pone.0277465.s004.tif]
