# Supplementary material for: Mobility in informal settlements during a public lockdown: A case study in South Africa
Source: PLoS One. 2022 Dec 22;17(12):e0277465. doi: 10.1371/journal.pone.0277465 (PMC9778567; doi:10.1371/journal.pone.0277465)
Supplement: S1 Table — (PDF) [file pone.0277465.s005.pdf]

**S1 Table. Average five-minute motion by week of the study.**

| | Feb 14-May 14 Data | | Feb 14-Jun 18 Data | |
|---|---|---|---|---|
| | (1) | (2) | (3) | (4) |
| Week 1: Feb.14-20 (constant) | 1.698*** | 1.340*** | 2.121*** | 1.555*** |
| | (0.012) | (0.020) | (0.025) | (0.126) |
| Week 2: Feb.21-27 | 0.137*** | 0.627*** | 0.232*** | 0.571*** |
| | (0.017) | (0.032) | (0.037) | (0.188) |
| Week 3: Feb.28-Mar.5 | 0.139*** | 0.242*** | 0.079** | 0.109 |
| | (0.017) | (0.029) | (0.034) | (0.130) |
| Week 4: Mar.6-12 (WHO Announcement) | -0.176*** | 0.108*** | -0.278*** | 0.168 |
| | (0.016) | (0.029) | (0.032) | (0.254) |
| Week 5: Mar.13-19 (State of Disaster) | -0.256*** | 0.154*** | -0.546*** | -0.022 |
| | (0.016) | (0.029) | (0.033) | (0.129) |
| Week 6: Mar.20-26 (Lockdown Announced) | -0.427*** | -0.343*** | -0.613*** | -0.106 |
| | (0.015) | (0.025) | (0.030) | (0.275) |
| Week 7: Mar.27-Apr.2 (Level 5 Begins) | -0.550*** | -0.286*** | -0.639*** | -0.518*** |
| | (0.015) | (0.026) | (0.032) | (0.128) |
| Week 8: Apr.3-9 | -0.781*** | -0.784*** | -0.921*** | -0.946*** |
| | (0.014) | (0.022) | (0.030) | (0.135) |
| Week 9: Apr.10-16 | -0.854*** | -0.845*** | -1.086*** | -1.081*** |
| | (0.014) | (0.022) | (0.029) | (0.126) |
| Week 10: Apr.17-23 | -0.757*** | -0.715*** | -0.829*** | -0.661** |
| | (0.014) | (0.023) | (0.031) | (0.267) |
| Week 11: Apr.24-30 | -0.707*** | -0.688*** | -0.717*** | -0.748*** |
| | (0.014) | (0.023) | (0.032) | (0.217) |
| Week 12: May 1-7 (L4 Begins) | -0.746*** | -0.837*** | -0.974*** | -1.080*** |
| | (0.014) | (0.022) | (0.029) | (0.126) |
| Week 13: May 8-14 | -0.777*** | -0.817*** | -0.948*** | -1.081*** |
| | (0.014) | (0.022) | (0.029) | (0.126) |
| Week 14: May 15-21 | | | -0.963*** | -1.146*** |
| | | | (0.030) | (0.126) |
| Week 15: May 22-28 | | | -0.864*** | -1.067*** |
| | | | (0.031) | (0.126) |
| Week 16: May 29-Jun.4 (L3 Begins) | | | -0.941*** | -1.074*** |
| | | | (0.029) | (0.126) |
| Week 17: Jun.5-11 | | | -0.659*** | -0.867*** |
| | | | (0.032) | (0.127) |
| Week 18: Jun.12-18 | | | -0.587*** | -0.804*** |
| | | | (0.033) | (0.127) |
| Type: | Paths | Compounds | Paths | Compounds |
| Observations | 1,074,445 | 472,000 | 476,102 | 436,683 |
| Adjusted $R^2$ | 0.017 | 0.023 | 0.014 | 0.001 |

*Note:* Left out group is week 1 in each data set (Feb 14 – Feb 20, 2020). The results in columns 1 and 2 include data from 60 path sensors and 26 compound sensors; the results in columns 3 and 4 include data from 21 path sensors and 18 compound sensors. Robust standard errors are in parentheses. *p<0.1; **p<0.05; ***p <0.01
