# Supplementary material for: Mobility in informal settlements during a public lockdown: A case study in South Africa
Source: PLoS One. 2022 Dec 22;17(12):e0277465. doi: 10.1371/journal.pone.0277465 (PMC9778567; doi:10.1371/journal.pone.0277465)
Supplement: S2 Table — (PDF) [file pone.0277465.s006.pdf]

S2 Table. Effect of South Africa's lockdown on activity.

	Paths				Paths (Inc. June)				Compounds				Compounds (Inc. June)			
	(1)	(2)	(3)	(4)	(5)	(6)	(7)	(8)	(9)	(10)	(11)	(12)	(13)	(14)	(15)	(16)
Lockdown (=1)	-0.643***	-0.658***			-0.668***	-0.714***			-0.841***	-0.844***			-1.045***	-1.101***		
	(0.005)	(0.005)			(0.011)	(0.010)			(0.010)	(0.009)			(0.067)	(0.074)		
Level 5			-0.633***	-0.650***			-0.662***	-0.706***			-0.795***	-0.796***			-0.910***	-0.872***
			(0.006)	(0.005)			(0.012)	(0.012)			(0.010)	(0.010)			(0.086)	(0.085)
Level 4			-0.665***	-0.678***			-0.774***	-0.818***			-0.959***	-0.965***			-1.215***	-1.341***
			(0.007)	(0.007)			(0.012)	(0.012)			(0.011)	(0.011)			(0.063)	(0.085)
Level 3							-0.497***	-0.551***							-1.000***	-1.117***
							(0.015)	(0.014)							(0.064)	(0.086)
Constant	1.602***	3.284***	1.602***	3.284***	1.944***	3.216***	1.944***	3.215***	1.471***	1.161***	1.471***	1.162***	1.674***	1.355***	1.674***	1.353***
	(0.005)	(0.033)	(0.005)	(0.033)	(0.009)	(0.028)	(0.009)	(0.028)	(0.009)	(0.022)	(0.009)	(0.022)	(0.063)	(0.051)	(0.063)	(0.051)
Sensor FE	No	Yes	No	Yes	No	Yes	No	Yes	No	Yes	No	Yes	No	Yes	No	Yes
Mean	1.254	1.254	1.254	1.254	1.49	1.49	1.49	1.49	1.02	1.02	1.02	1.02	0.98	0.98	0.98	0.98
L5 v. L4			-0.03***<br>(0.006)								-0.16***<br>(0.009)					
L4 v. L3							-0.28***<br>(0.014)								-0.22***<br>(0.011)	
N	1,074,445	1,074,445	1,074,445	1,074,445	476,102	476,102	476,102	476,102	472,000	472,000	472,000	472,000	436,683	436,683	436,683	436,683
Adj. R^2	0.014	0.098	0.014	0.098	0.010	0.105	0.010	0.105	0.017	0.096	0.017	0.096	0.001	0.002	0.001	0.002

Note: Left out group is "level 0" (pre-lockdown) in all specifications. Results in columns 1-4 and 9-12 include data until May 14, 2020 from 60 path sensors and 26 compound sensors, respectively. Results in columns 5-8 and 13-16 include data until June 18, 2020 from 21 path sensors and 18 compound sensors. Robust standard errors are in parentheses in the main table. Welch's Two-Sample t-test of difference in means between Level 5 and 4 and between Level 4 and 3 reported in bottom panel and the standard errors are in parentheses. *p<0.1; **p<0.05; ***p <0.01
