# Supplementary material for: Mobility in informal settlements during a public lockdown: A case study in South Africa
Source: PLoS One. 2022 Dec 22;17(12):e0277465. doi: 10.1371/journal.pone.0277465 (PMC9778567; doi:10.1371/journal.pone.0277465)
Supplement: S3 Table — (PDF) [file pone.0277465.s007.pdf]

S3 Table. The Oxford Stringency Index (SI) and nighttime activity.

	Paths		Compounds	
	(1)	(2)	(3)	(4)
SA Stringency Index (cont.)	-0.009***		-0.012***	
	(0.0001)		(0.0001)	
Level: 13.89		-0.310***		-0.290***
		(0.010)		(0.017)
Level: 19.44		0.108***		0.855***
		(0.026)		(0.045)
Level: 38.89		-0.256***		0.182***
		(0.019)		(0.033)
Level: 55.56		-0.603***		-0.747***
		(0.010)		(0.018)
Level: 84.26		-0.870***		-1.140***
		(0.009)		(0.015)
Level: 87.96		-0.823***		-0.946***
		(0.007)		(0.012)
Constant	1.777***	1.806***	1.675***	1.652***
	(0.005)	(0.006)	(0.008)	(0.010)
Mean	1.254	1.254	1.02	1.02
Observations	1,074,445	1,074,445	472,000	472,000
Adjusted R^2	0.016	0.017	0.018	0.021

Note: In models 2 and 4, the index is converted to a categorical variable to better understand how activity responds to changes in levels. Robust standard errors are in parentheses. Results that include sensor fixed effects can be provided by the authors upon request. *p<0.1; **p<0.05; ***p <0.01
