# Supplementary material for: Mobility in informal settlements during a public lockdown: A case study in South Africa
Source: PLoS One. 2022 Dec 22;17(12):e0277465. doi: 10.1371/journal.pone.0277465 (PMC9778567; doi:10.1371/journal.pone.0277465)
Supplement: S4 Table — (PDF) [file pone.0277465.s008.pdf]

S4 Table. Effect of lockdown by day of week.

	Paths		Compounds	
	(1)	(2)	(3)	(4)
Lockdown (=1)	-0.720*** (0.015)	-0.736*** (0.014)	-1.039*** (0.026)	-1.042*** (0.024)
Tuesday	-0.265*** (0.016)	-0.266*** (0.016)	-0.306*** (0.031)	-0.306*** (0.030)
Wednesday	-0.305*** (0.016)	-0.304*** (0.015)	-0.453*** (0.031)	-0.453*** (0.029)
Thursday	-0.169*** (0.017)	-0.168*** (0.016)	-0.187*** (0.032)	-0.187*** (0.030)
Friday	0.132*** (0.018)	0.132*** (0.017)	-0.065** (0.033)	-0.065** (0.031)
Saturday	0.199*** (0.017)	0.200*** (0.017)	-0.143*** (0.032)	-0.143*** (0.030)
Sunday	0.185*** (0.018)	0.187*** (0.017)	0.226*** (0.035)	0.226*** (0.033)
Tuesday*Lockdown	0.232*** (0.020)	0.233*** (0.019)	0.326*** (0.035)	0.326*** (0.033)
Wednesday*Lockdown	0.384*** (0.020)	0.383*** (0.019)	0.675*** (0.035)	0.674*** (0.033)
Thursday*Lockdown	0.198*** (0.020)	0.197*** (0.019)	0.224*** (0.036)	0.222*** (0.034)
Friday*Lockdown	0.039* (0.021)	0.040* (0.020)	0.258*** (0.037)	0.260*** (0.035)
Saturday*Lockdown	-0.093*** (0.021)	-0.090*** (0.020)	0.133*** (0.035)	0.135*** (0.034)
Sunday*Lockdown	-0.219*** (0.021)	-0.217*** (0.020)	-0.229*** (0.039)	-0.228*** (0.036)
Constant	1.634*** (0.012)	3.315*** (0.035)	1.604*** (0.023)	1.294*** (0.030)
Sensor Fixed Effects	No	Yes	No	Yes
Mean	1.254	1.254	1.02	1.02
Observations	1,074,445	1,074,445	472,000	472,000
Adjusted R^2	0.017	0.101	0.019	0.098

Note: Left out group is Monday in all specifications. Robust standard errors are in parentheses.
*p<0.1; **p<0.05; ***p<0.01
