# Supplementary material for: Mobility in informal settlements during a public lockdown: A case study in South Africa
Source: PLoS One. 2022 Dec 22;17(12):e0277465. doi: 10.1371/journal.pone.0277465 (PMC9778567; doi:10.1371/journal.pone.0277465)
Supplement: S5 Table — (PDF) [file pone.0277465.s009.pdf]

S5 Table. Effect of lockdown by hour.

	Paths		Compounds	
	(1)	(2)	(3)	(4)
Lockdown (=1)	-1.097*** (0.034)	-1.112*** (0.031)	-2.722*** (0.067)	-2.724*** (0.064)
7:00 pm	-0.785*** (0.036)	-0.785*** (0.033)	-1.669*** (0.074)	-1.669*** (0.070)
8:00 pm	-2.293*** (0.033)	-2.293*** (0.031)	-3.193*** (0.068)	-3.193*** (0.064)
9:00 pm	-3.461*** (0.031)	-3.461*** (0.029)	-4.182*** (0.065)	-4.182*** (0.061)
10:00 pm	-4.350*** (0.031)	-4.350*** (0.029)	-4.831*** (0.063)	-4.831*** (0.060)
11:00 pm	-4.756*** (0.030)	-4.756*** (0.028)	-5.043*** (0.062)	-5.043*** (0.059)
12:00 am	-4.964*** (0.030)	-4.965*** (0.028)	-5.178*** (0.062)	-5.177*** (0.059)
1:00 am	-5.074*** (0.029)	-5.075*** (0.028)	-5.252*** (0.061)	-5.252*** (0.059)
2:00 am	-5.142*** (0.029)	-5.143*** (0.027)	-5.334*** (0.061)	-5.334*** (0.058)
3:00 am	-5.172*** (0.029)	-5.173*** (0.027)	-5.350*** (0.061)	-5.350*** (0.058)
4:00 am	-5.162*** (0.029)	-5.162*** (0.027)	-5.414*** (0.060)	-5.414*** (0.057)
5:00 am	-4.890*** (0.029)	-4.890*** (0.027)	-5.188*** (0.060)	-5.188*** (0.058)
6:00 am	-4.473*** (0.029)	-4.473*** (0.027)	-5.060*** (0.060)	-5.060*** (0.058)
7:00 am	-3.522*** (0.032)	-3.523*** (0.030)	-3.920*** (0.068)	-3.920*** (0.066)
7pm*Lockdown	-0.590*** (0.043)	-0.590*** (0.040)	0.344*** (0.084)	0.344*** (0.080)
8pm*Lockdown	-0.391*** (0.040)	-0.391*** (0.037)	1.130*** (0.078)	1.129*** (0.073)
9pm*Lockdown	0.081** (0.038)	0.081** (0.035)	1.775*** (0.074)	1.774*** (0.070)
10pm*Lockdown	0.515*** (0.037)	0.515*** (0.034)	2.192*** (0.073)	2.192*** (0.069)
11pm*Lockdown	0.745*** (0.036)	0.745*** (0.034)	2.290*** (0.072)	2.289*** (0.068)
12am*Lockdown	0.839*** (0.036)	0.840*** (0.034)	2.370*** (0.071)	2.369*** (0.068)
1am*Lockdown	0.906*** (0.035)	0.907*** (0.033)	2.443*** (0.071)	2.443*** (0.068)
2am*Lockdown	0.930*** (0.035)	0.931*** (0.033)	2.470*** (0.070)	2.470*** (0.067)
3am*Lockdown	0.971*** (0.035)	0.972*** (0.033)	2.514*** (0.070)	2.513*** (0.067)
4am*Lockdown	0.962*** (0.035)	0.962*** (0.033)	2.563*** (0.069)	2.562*** (0.066)
5am*Lockdown	0.794*** (0.035)	0.794*** (0.033)	2.416*** (0.070)	2.416*** (0.067)
6am*Lockdown	0.533*** (0.036)	0.533*** (0.033)	2.333*** (0.070)	2.334*** (0.067)
7am*Lockdown	0.070* (0.038)	0.070** (0.036)	1.487*** (0.078)	1.487*** (0.075)
Constant	5.461*** (0.028)	7.144*** (0.037)	5.729*** (0.058)	5.420*** (0.059)
Sensor Fixed Effects	No	Yes	No	Yes
Mean	1.254	1.254	1.02	1.02
Observations	1,074,445	1,074,445	472,000	472,000
Adjusted R^2	0.286	0.370	0.159	0.237

Note: Left-out group is “level 0” (before lockdown) and 0:00 (midnight). Robust standard errors are in parentheses. *p<0.1; **p<0.05; ***p<0.01
