# Supplementary material for: Mobility in informal settlements during a public lockdown: A case study in South Africa
Source: PLoS One. 2022 Dec 22;17(12):e0277465. doi: 10.1371/journal.pone.0277465 (PMC9778567; doi:10.1371/journal.pone.0277465)
Supplement: S6 Table — (PDF) [file pone.0277465.s010.pdf]

S6 Table. Robustness Checks.

	Five-Minute Motion		Mean Hourly Motion	
	Paths (no 6pm/7am)	Compounds (no 6pm/7am)	Paths	Compounds
	(1)	(2)	(3)	(4)
Lockdown (=1)	-0.573***	-0.652***	-6.564***	-9.610***
	(0.005)	(0.009)	(0.283)	(0.657)
Temperature			0.825***	0.768***
			(0.063)	(0.139)
Constant	1.252***	1.088***	55.445***	50.625***
	(0.004)	(0.008)	(1.574)	(3.590)
Hour Dummies	No	No	Yes	Yes
Weekday Dummies	No	No	Yes	Yes
Mean	0.942	0.739	1.254	1.02
Observations	920,924	404,572	75,578	32,981
Adjusted R^2	0.016	0.015	0.401	0.181

Note: We do not control for sensor fixed effects in these robustness checks, but the results including sensor fixed effects can be provided by the authors upon request. Robust standard errors in parentheses. *p < 0.1; **p < 0.05; ***p < 0.01
